# Approach to concurrent inguinal hernias during laparoscopic appendectomy for early appendicitis

**DOI:** 10.1007/s00423-024-03269-0

**Published:** 2024-03-04

**Authors:** Sabriye Dayı, Meryem Anayurt, Fatma Çınar, İsmet Kırmacı, Serpil Sancar

**Affiliations:** Department of Pediatric Surgery, University of Health Sciences, Bursa City Hospital, Bursa, Türkiye

**Keywords:** Appendicitis, Inguinal hernia, Concurrent, Laparoscopy

## Abstract

**Purpose:**

Concurrent inguinal hernia can be a challenge for a pediatric surgeon during a laparoscopic appendectomy in children. We aimed to present our approach to inguinal hernias seen during laparoscopic appendectomy.

**Methods:**

In our pediatric surgery clinic between July 2019 and December 2022, the records of patients aged 0–18 who underwent laparoscopic appendectomy were retrospectively investigated. The clinical findings, surgical procedure, and results of intervened inguinal hernia cases during laparoscopic appendectomy were evaluated.

**Results:**

Between the study dates, 293 laparoscopic appendectomies were performed in our clinic. Patent processus vaginalis was observed in 5 (1.7%) cases. Laparoscopic hernia repair was performed with the Burnia technique in 2 girls. In one case, the omentum was herniated and adhered to the hernia sac seen during laparoscopy. In another case, swelling in the groin occurred during the introduction of air into the abdomen at the beginning of the operation. Patent processus vaginalis seen in 3 asymptomatic cases was not intervened. There were no postoperative complications or recurrences in the two patients who underwent hernia repair.

**Conclusion:**

Hernia repair with the Burnia technique might be safely performed in symptomatic inguinal hernia cases seen during laparoscopic appendectomy for early appendicitis.

## Introduction

Appendicitis and inguinal hernia are the most common pediatric surgery operations. In the pediatric population, the incidence of appendicitis is reported to be 1 in 1000 [1], and the incidence of inguinal hernia is 0.8–12% [2–4].

In recent years, appendicitis operations in children have started to be performed widely with the laparoscopy method. Due to the high incidence of inguinal hernia in children, it may be encountered incidentally during laparoscopic appendectomy. Ditsatham et al. [5] calculated the probability of the two conditions occurring together at approximately 9 per 1,000,000 patients, based on studies by Lee et al. [6] and van den Heuvel et al. [7]. Li Ye et al. [8] found the frequency of concurrent inguinal hernia (including patent processus vaginalis) to be 5.7% in laparoscopic appendicitis in a multicentric study conducted in children. Using laparoscopy as a minimally invasive procedure in appendicitis surgery may have increased the likelihood of a coincidental diagnosis of other diseases.

When we searched the literature, there needed to be more studies about the approach to detect inguinal hernias incidentally. Although Li Ye et al. [8] reported that performing a laparoscopic appendectomy and inguinal hernia operation in children is safe and effective. However, there has yet to be a complete consensus on whether it would be better to intervene in these two simultaneous conditions simultaneously or postpone them. In addition, it is unclear which cases of appendicitis and what kind of inguinal hernia surgery can be performed.

We retrospectively reviewed our laparoscopic appendectomy cases in our clinic to find an answer to whether it is safe to delay or simultaneously perform inguinal hernia repair encountered in appendicitis surgery.

## Method

Our study was carried out in the pediatric surgery clinic of our institution between July 2019 and December 2022. Patients with incidentally detected processus vaginalis during laparoscopic appendectomy were evaluated. Patients with swelling in the inguinal region by external observation during laparoscopy or herniation of the intra-abdominal organs into the inguinal canal were evaluated as symptomatic inguinal hernia, and hernia repair was decided.

### Surgical method

Laparoscopy was performed under general anesthesia with three port 30-degree optics 10–12-mmHg pneumoperitoneum. Appendectomy was performed with an intracorporeal knot by 2/0 silk suture. Laparoscopic hernia repair was performed with the Burnia method. In the Burnia method, the hernia sac is captured by entering the inguinal canal, retracted, and cauterized by carefully protecting the surrounding tissues.

Our study evaluated demographic characteristics, clinical and operative findings, and postoperative follow-up results of patients who underwent laparoscopic appendectomy and simultaneous hernia repair.

## Results

Our clinic performed 293 laparoscopic appendectomies within the study period. Patent processus vaginalis was observed in 5 (1.7%) cases (3 boys and 2 girls). Laparoscopic hernia repair was performed with the Burnia technique in 2 girls (0.6%). In one case, in a 10-year-old girl, the omentum was herniated and adhered to the right inguinal hernia sac seen during laparoscopy. In another case, a 10-year-old girl, swelling in the left groin occurred during the introduction of air into the abdomen at the beginning of the operation.

**Fig. 1:**
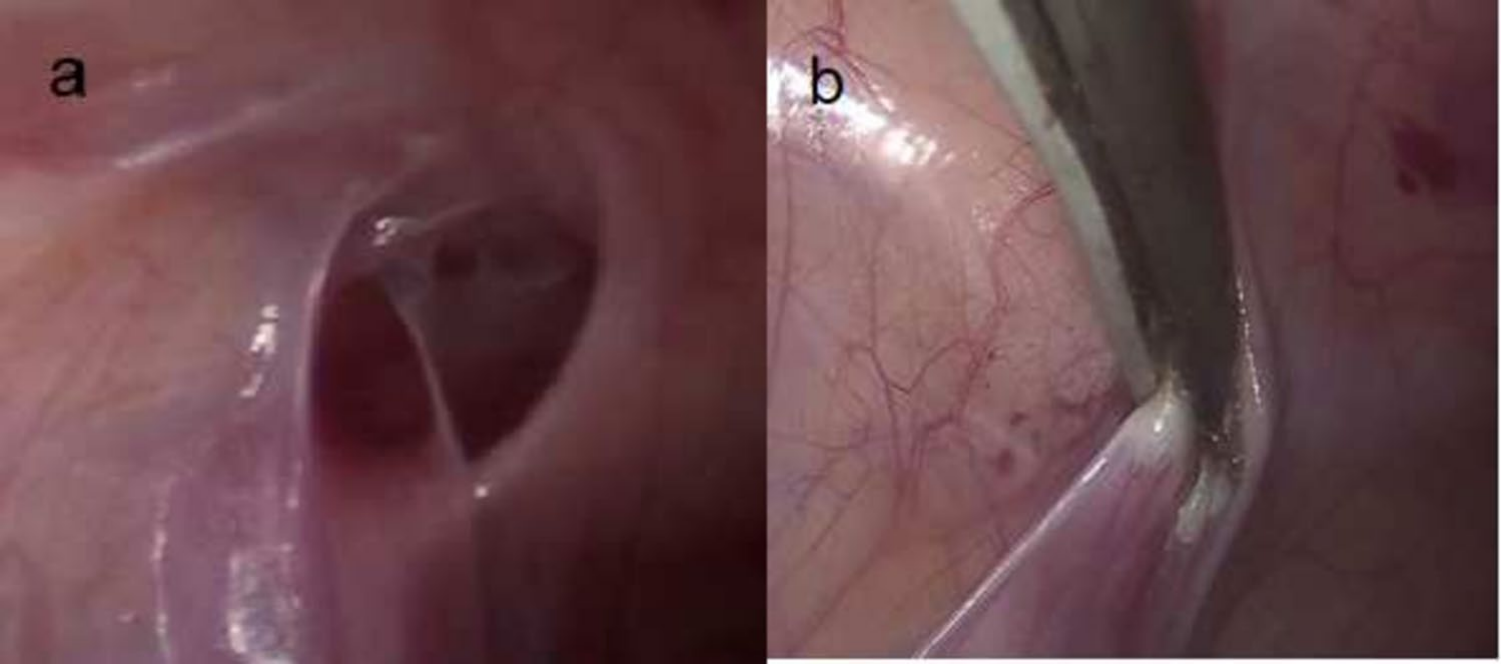
Surgical intervention for inguinal hernia detected incidentally during laparoscopic appendectomy surgery. **a**. Left inguinal hernia in a 7-year-old girl; **b**. laparoscopic hernia repair by cauterization of the left inguinal hernia sac (Burnia method)

The first patient undergoing hernia repair had phlegmonous appendicitis, and the second one had acute appendicitis. Laparoscopic appendectomies were performed, and then inguinal hernias were repaired with the Burnia technique (Fig. 1) in the same session. There were no postoperative complications in the 2 patients who underwent hernia repair. No inguinal hernia recurrence was detected in the 2-year follow-up of these 2 cases. Patent processus vaginalis seen in 3 asymptomatic cases was not intervened.

## Discussion

With the increase in the variety of minimally invasive interventions in operations performed on children, the rate of disease detection is increasing simultaneously. During laparoscopic appendectomy in children, an inguinal hernia can be detected, and patent processus vaginalis can be seen. In the literature, this rate was 5.7% [8] when the patent processus vaginalis was considered, while it was 1.7% in our study. Intra-abdominal organ herniation into the inguinal canal or swelling in the inguinal region from the outside was considered a symptomatic inguinal hernia, and simultaneous hernia repair was performed in 0.6% of cases in our study. This high rate can be explained as an additional contribution of laparoscopy. Studies to be carried out by increasing the study population will give more precise rates.

Simultaneous surgery has traditionally been avoided as there are concerns that it may increase surgical site infections and lead to the recurrence of inguinal hernia [8]. In a multicentric study by Li et al. [8], they presented the results of simultaneous laparoscopic appendectomy and inguinal hernia repair. They stated that simultaneous laparoscopic inguinal hernia and appendectomy are safe, effective, and feasible when the indications and contraindications in the operations are closely monitored. Concurrent surgeries presented in many studies in the literature are related to Amyand’s hernia and appendectomy [9, 10]. Ditsatham et al. [5] used two different incisions in a patient they presented, and appendectomy with laparotomy and inguinal hernia repair with inguinal incision were performed in the same session. Laparoscopy allows simultaneous appendectomy and hernia repair in the same session without an additional incision. Therefore, laparoscopy is more advantageous than open surgery in patients undergoing simultaneous repair. In recent years, almost all appendectomies have been performed by a laparoscopic method in our clinic. In our study, we preferred to perform simultaneous hernia repair in patients considering hernia repair.

The inguinal hernia repair used by Li et al. [8] was silk suturing of the internal ring. However, whether suturing increases recurrence due to intra-abdominal appendicitis inflammation is not known. Li et al. [8] found recurrence in 1 patient in a large study group. We used the Burnia technique [11, 12] in our patients with inguinal hernia. The Burnia technique used in inguinal hernia is an easy method for laparoscopic appendectomy. This technique was used in 2 girls in our patients, and no recurrence was observed during the 2-year follow-up period. No male inguinal hernia was detected in our patient group. The Burnia technique can be used with caution in boys because of the proximity of the spermatic cord elements to the hernia sac.

Performing both surgeries at the same time is cost-effective. Another advantage is that the child and family do not experience the stress of surgery twice. Before the operation, it is crucial to question the history of the sick child, conduct a careful examination, and obtain appropriate consent from the family.

## Conclusion

Laparoscopic appendectomy contributes to the diagnosis of concomitant patent processus vaginalis. Simultaneous hernia repair can be safely performed with the Burnia method in symptomatic inguinal hernia cases detected incidentally during laparoscopic hernia repair. However, additional studies are needed to be done safely in the advanced stages of appendicitis inflammation and when there is an intra-abdominal abscess.

## Data Availability

The data supporting this study’s findings, which were used under permission from the University of Health Sciences, Bursa City Hospital, are available from the authors.
